# Mobilizing Collective Action for Public Health Approaches to Dementia Education

**DOI:** 10.1093/geroni/igaa057.006

**Published:** 2020-12-16

**Authors:** Jennifer Severance, Susanna Luk-Jones, Griffin Melissa, Glenda Redeemer

**Affiliations:** 1 UNT Health Science Center, Fort Worth, Texas, United States; 2 Alzheimer's Association, North Central Texas Chapter, Fort Worth, Texas, United States; 3 Tarrant County Public Health, Fort Worth, Texas, United States

## Abstract

Addressing increasing rates of Alzheimer’s disease and related dementias (ADRD) requires public health approaches including prevention, early detection and diagnosis, and outreach to low-income and minority communities facing higher risk and adverse health and economic outcomes. Communities are seeking ways to enhance cross-sector collaboration and overcome underdeveloped relationships and fragmentation that are barriers to effective public health responses. In this exploratory study, we evaluated outcomes of a community-wide effort to mobilize systems-level changes, build public awareness, and increase access to early detection services. A community-based organization, public health department, and academic institution in North Texas partnered to expand ADRD education programs and outreach for underserved communities. Nineteen community health workers were trained to provide brain health and ADRD education programs and refer to financial, legal, and social resources. Through collective action, 371 participants attended 26 education sessions delivered in English and Spanish. Forty-five percent of participants identified as non-white and 61% reported low educational attainment. Participants (n=314) completed post-surveys. As a result of training, 89% of trainees could recognize common warning signs of Alzheimer’s disease, 86% understood the importance of early detection and diagnosis, and 96% knew activities promoting cognitive health. Findings revealed strategies to increase collective action such as sharing data, establishing referral methods, and adopting dementia-friendly and age-friendly frameworks. Results show that collective action has the potential to build a community’s capacity for targeted ADRD education and improve access to early detection and brain health education for at-risk populations.

